# Physical Activity and Cognitive Functioning

**DOI:** 10.3390/medicina60020216

**Published:** 2024-01-26

**Authors:** Francesca Latino, Francesco Tafuri

**Affiliations:** 1 Department of Human Science, Educational and Sport, Pegaso University, 80143 Naples, Italy; 2 Heracle Lab Research in Educational Neuroscience, Niccolò Cusano University, 00166 Rome, Italy; francesco.tafuri@unicusano.it

**Keywords:** neuroscience, able-bodied, disabled children, learning, physical activity, cognitive function

## Abstract

Neuroscience applied to motor activity is a growing area that aims to understand the effects of motor activity on the structures and functions of the Central Nervous System. Attention has been paid to this multidisciplinary field of investigation by the scientific community both because it is of great importance in the treatment of many chronic diseases and because of its potential applications in the Movement Sciences. Motor activity during a developmental age is, in fact, an indispensable tool for the physical and mental growth of children, both able-bodied and disabled. Through movement, individuals can improve their physical efficiency and promote their own better health, establish relationships with the environment and others, express themselves and their emotions, form their identity and develop cognitive processes. This literature review aims, therefore, to highlight how an adequate practice of motor activity offers extraordinary possibilities for everyone in relation to learning, from the perspective of an integral development of the person, and, consequently, can raise the awareness of those involved in the training and growth, especially the youngest, towards the educational value of motor and sports activities. According to this review, and in line with the modern neuroscientific approach toward the relationships between motor activities and cognitive functions, it is possible to claim that hypokinesia tends to inhibit learning. Therefore, it now seems more topical than ever to draw attention to the need to introduce working proposals that integrate brain-based motor activity programs into the school curriculum.

## 1. Introduction

Motor and sporting activity is always recognized for its many benefits for everyone. The positive effects on the psycho-physical well-being of the individual have led to the proposal of motor activity as a true therapy, often an alternative to medication. Over the past few decades, numerous studies have shown that it can also induce significant cognitive and neuroprotective benefits, especially on processes involving learning abilities and memory [1,2,3].

According to a conceptualization model proposed by many authors [4,5,6], this interdependence is mainly related to the changes that occur in brain function and structure as a consequence of motor activity. In recent years, research in this field has evolved considerably through studies born out of the need to better understand the multifaceted relationship between motor activity and cognitive health. Advances in neuroscience and neuroimaging techniques have enabled researchers to better understand the effects of motor activity on cognitive functions by investigating its physiological and molecular mechanisms [7]. Research conducted in the different areas of interest concerning motor activity has helped us to define this line of investigation and to identify some preliminary underlying mechanisms. A deeper understanding of the components that are related to cognitive function and that may be amenable to intervention has thus been obtained [8]. Investigations into the influence of motor activity on cognitive functioning have proposed several mechanisms that could explain this relationship [9]. Numerous studies published in recent years on this subject have mainly concluded that motor activity is able to bring about a series of biological and structural modifications and adaptations that allow brain cells to create new connections in different cortical areas. Motor activity, in fact, is able to trigger a cascade of neurochemical growth factors capable of changing the entire brain’s structure. This reflects the brain’s ability to adapt to the various cognitive challenges that it faces [10]. Studies using imaging techniques have thus demonstrated that movement can stimulate angiogenesis, i.e., the development of new blood vessels from existing ones, neurogenesis, i.e., the presence of undifferentiated neuronal cells capable of self-renewal and differentiation into different neuronal lines, and improve synaptic plasticity, i.e., the nervous system’s ability to modify the efficiency of the functioning of connections between neurons, to eliminate some and establish new ones [11,12]. Motor and sporting activity conducted on a constant and regular basis is, therefore, capable of favorably influencing cognitive processes as:

1. It facilitates neural development;
2. It increases the concentration of synapses between neurons;
3. It promotes vascular circulation in the cerebral cortex;
4. It promotes the synthesis of neurotransmitters and neurotrophies that trigger the processes of neurogenesis, angiogenesis and neuroplasticity.

Although studies conducted on the human brain are still limited compared to those carried out on animal test subjects, there is now ample evidence that, contrary to past belief that the adult brain remains static after prenatal and neonatal development, new neurons can be generated through a process known as neurogenesis [13]. In recent decades, in fact, numerous experimental trials have shown that neurogenesis can also occur in the adult brain, particularly at the level of the olfactory bulb and in the hippocampus, where new undifferentiated neuronal stem cells give rise to neural progenitor cells. It is precisely the hippocampus, located in the temporal lobe and embedded in the limbic system, that plays a key role in learning, spatial orientation and memory, as well as being a fundamental area in the cognitive processes related to the construction of our identity and in the adaptive capacities of human beings. In fact, as people age, there are several changes in the brain, to a slight degree, that affect cognitive, behavioral and emotional functions. According to the definition provided by the American Association of Psychologists (APA) in 1987, “chronic-progressive cognitive impairment is a brain disease that involves the impairment of cognitive functions such as to impair the possibility of living independently. Cognitive symptoms are almost always associated with alterations in personality and behavior that vary in entity from individual to individual. In addition, there is a progressive alteration of the functional status.” Cognitive symptoms are different [14]. They vary depending on the diagnosis and the portion of brain tissue and nerve cells affected. The most common cognitive symptoms may be memory impairment, language difficulties, reduced attention, slowed logical reasoning, a progressive reduction in perceptual skills and a reduction in movement skills.

The improvement of physical performance has a protective effect against cognitive decline in the course of aging [15]. Physical activity is considered a determining factor in the health of adults and especially in those over 60 years of age. It helps slow down the decline in cognitive performance. An adequate program of physical activity in the elderly improves brain function by modifying the passage across the blood–brain barrier of chemicals important for mood and nerve transmission [16]. The favorable effects of physical exercise are then amplified if there is a simultaneous involvement of the elderly in social and productive activities. This happens independently of the improvement in cardio-pulmonary performance, suggesting that physical activity can bring benefits in terms of survival and quality of life. It should be emphasized that the positive effect, although widespread, is not generic, but mainly oriented towards executive functions and the speed of information processing [17]. The protective effect of physical activity on the cognitive function of the elderly has several explanations. First of all, it has a well-established effect on reducing cardio- and cerebrovascular risk, arterial hypertension and diabetes mellitus, all relevant factors in the pathogenesis of several forms of cognitive decline, including Alzheimer’s disease and vascular dementia. Furthermore, physical activity seems to have a direct effect at the biochemical and structural level (increased production of neuronal growth factors, such as BNDF, serotonin and IGF-1; increased neuroplasticity in animal studies; increased hippocampal volume and dentate gyrus in studies on human subjects; and a reduction in the accumulation of neurodegenerative products such as beta-amyloid and tau protein) [18].

Therefore, this contribution intends to explore, through a review of the scientific research in the literature, the link between motor and sport activity and learning processes, based on the assumption that motor activity can be an important mediating factor capable of making brain functions more efficient, plastic and adaptable, due to positive modifications that trigger changes in neurogenesis, synaptic plastic capacity and neuronal proliferation.

## 2. Search Strategy, Identification of the Studies and Study Characteristics

This literature review presents an overview of studies focused on how an adequate practice of motor activity offers possibilities for everyone in relation to learning, from the perspective of an integral development of the person.

In order to identify the studies four databases were used: Google Scholar, Pubmed, Web of Science and Cochrane Library. The following Boolean search syntax was applied to identify the studies: “((Physical activity” or “physical exercise”) and (“cognitive function” or “cognition”)”/“(“learning”) and (“academic achievement” or “academic performance”))”. Then, different filters were applied: text availability, full text; species, humans and animals; languages, English; period, last thirty years. Lastly, further selection has been provided due to the use of inclusion and exclusion criteria, such as:

(i) English-language publications;
(ii) A time interval of studies, between 1990 and 2023;
(iii) Physical activity or exercise as a tool for the improvement of cognitive function;
(iv) Study design: randomized controlled trials.

Studies were excluded if they did not meet the inclusion criteria, or due to their lack of focus on physical activity or exercise interventions.

## 3. The Effects of Motor and Sport Activity on Neuroplasticity

What has been discussed so far allows us to understand how motor activity is able to influence and modify neuronal processes. Indeed, there is a great deal of scientific evidence demonstrating that motor activity leads to an increase in cerebral plasticity, especially in the hippocampus [19]. In years past, the scientific community believed that the Central Nervous System (CNS) was structurally stable immediately after birth and that only a few changes could take place within it during the course of a person’s life. The different brain areas were thus represented as predefined and immutable structures. This belief made the brain an organism that, once it had reached its full development, became static and destined to a gradual, yet irreversible, decline [20,21]. However, over the past few decades, with the advancement of imaging techniques, this dogma began to s to the belief that the human brain can manifest plasticity phenomena at all stages of life. The human brain is not, in fact, made up of fixed and immutable neural circuits, but the brain’s synaptic network and the structures connected to it are capable of actively reorganizing themselves through practice and experience, reprogramming their neural networks [22]. Neuroplasticity is the brain’s ability to change its structure and functioning based on lived experiences [23], and thus also change in relation to motor activity.

Neuroplasticity is a kind of structural and functional disposition of our nervous system to change as a result of stresses from the environment [24]; the richer and more diversified the stresses are, the more our brain will be able to adapt and modulate itself in response to them. Several studies have shown how motor activity conducted in an enriched environment causes structural modifications of the nervous system and behavioral changes through an increase in locomotor and exploratory activity, learning ability and especially spatial and problem-solving abilities. This condition is also capable of leading to a long-term enhancement of the hippocampus due to an activity-dependent increase in synaptic transmission [25,26]. This enrichment condition can also influence the expression of neurotrophins [27], especially BDNF (Brain-Derived Neurotrophic Factor), known as one of the main modulators of brain plasticity. The modulation of the release of neurotransmitters such as BNDF and their utilization by nerve structures is, in fact, related to the neuroplasticity induced by motor activity [28]. Neuroplasticity should also be considered a fundamental property from the perspective of normal neuronal and cognitive functioning, and not only a response to a pathological condition that results in a lesion; motor activity, experience, and learning situations are all factors that allow the synaptic connections of our CNS to be shaped [29,30]. Synaptic neuroplasticity is based on three fundamental principles that bring about changes in the functioning of neuronal circuits and modifications in brain reorganization. These principles are represented by the modifiability of functional and morphological synaptic transmission, by sprouting, i.e., a proliferation of axon collaterals with an associated formation of synapses, and by neurogenesis, i.e., the proliferation of certain populations of neurons that take on the characteristics of pluripotent stem cells.

Human studies have made it possible to describe four forms of brain plasticity. The first is Cortical Map Expansion, which is expressed in the ability of the primary cortices to expand their representation areas following the acquisition of skills or the repetition of an exercise [31]. In 1995, Elbert et al., studying a group of violinists, showed that in response to exercise, their left-hand finger representation area was larger than in the control group, as they represent a professional category that makes differentiated, as well as intensive, use of the left hand [32]. Cross-modal reassignment represents the second form of plasticity and implies the transfer of certain functions specific to a sensory modality to brain areas other than those originally assigned to them. The third form is Homologous Area Adaptation, which consists of the possibility of transferring the cognitive operations of an area in which a lesion has occurred to the contralateral homologous area of the cerebral hemisphere. The last form of neuroplasticity is expressed through the concept of masked compensation or vicariousness, and this represents the possibility of using alternative strategies and modalities to counteract and, indeed, partially vicariate the functions no longer performed by a damaged area, due to the recruitment of an intact cognitive system. From this point of view, motor activity would favor learning as it is able to trigger the release of a cascade of neurochemical factors capable of generating neurogenesis, especially at the level of the hippocampus [33], a region that, by its very nature, is the gateway to new memories essential for learning [34]. Neurogenesis is, in fact, a differentiation process that allows for the generation of new neurons, which takes place throughout life, in which neuronal stem cells evolve into mature neurons that will go on to form the brain’s network of interconnections [35]. This differentiation is facilitated by factors such as past experience, learning, physical and mental exercise. This is exactly what happens with exercise-mediated learning, which results in new stimuli that promote the creation of synaptic connections and, consequently, improved intellectual performance [36]. It seems, therefore, clear how the constant practice of motor and sporting activity nourishes and supports cognitive functions while at the same time fostering the development of a positive sense of self-efficacy, thus offering a positive feedback loop that supports learning. Neuroplasticity is, moreover, the basis of the concept of cognitive modifiability, of which it is the prerequisite and neurobiological counterpart, as well as its complex mediating phenomenon. Cognitive modifiability is the possibility of enhancing cognitive processes and internal brain resources that are not always exploited, but also the possibility of developing abilities that are absent or present to a reduced extent in an individual’s behavioral repertoire [37]. It describes the human organism’s tendency to allow itself to be shaped by its environment. Numerous studies underline how motor activity performed in adulthood allows for the activation of less-developed brain areas and the modification of neuronal maps [38]. It is precisely through movement, practice and experience that neuronal networks and related structures can actively reorganize themselves. When a new motor skill is acquired, the brain potential for neuroplasticity is set in motion. During the first exercise, the connections between the brain and the specific muscle group used to learn that skill are not optimized. With the repetition of the gesture, the neural pathway becomes more established and refined, so that more neurons and muscle fibers are recruited and coordinated within the correct motor pattern. The plastic changes that occur in the brain result in physical improvements related to the acquired ability. Motor activity allows the brain to function to its full potential, supporting neuronal multiplication and strengthening connections, thus leading to improved cognitive abilities [39]. Motor and sports activity programs are important as they prepare the brain for learning by placing it in the optimal conditions for learning [40]. Studies by Kleim and Jones [41] first, and Feuerstein et al. [42] later, show that there are essential criteria that underlie neuroplasticity and, thus, cognitive modifiability. The first criterion is the Activation effect, according to which, for there to be plasticity, learning interventions must be carried out in such a way as to stimulate specific brain functions. This is the essential criterion that cannot be disregarded, as it represents the potential of cognitive modifiability. Several studies have demonstrated the loss of certain neural connections and, consequently, of acquired abilities, when these brain functions are not exercised [43]. The second criterion is called the Specificity effect and is based on the principle that highly specific interventions are required to increase neuroplasticity and cognitive modifiability. The Repetition effect constitutes the third principle, whereby the repetition of the motor task is a prerequisite for changes to manifest themselves behaviorally. However, mere passive repetition is not sufficient to promote plasticity; there must be variability in the tasks proposed. The fourth condition is the Intensity effect, according to which the greater the frequency and time over which the function is stimulated, the more noticeable the change will also be behaviorally. The Persistence effect is based on the need for a certain level of constancy in relation to the exercise schedule, and is the fifth condition. The sixth criterion relates to the motivational dimension and is the Salience Effect; understanding the value of the intervention is the key to successful cognitive modifiability. The Optimal timing potential effect is the seventh criterion. According to this principle, change is correlated with age. Nevertheless, if the adult brain is subjected to appropriate stimulation, this does not constitute a barrier to learning. According to the eighth criterion, termed the Novelty effect, in order for any change to take place, the exercise of skills must incorporate new and increasingly complex elements. The Spread off effect is the ninth criterion and represents the possibility that the changes, and thus the new learning, may also extend to other functions not originally targeted by the intervention. The tenth criterion is the Selection effect and reflects the possibility that there may be interference caused by the new changes in other areas of operation. The eleventh is the Consciousness/awareness effect and concerns the need for the subject to be aware of the changes he or she undergoes as a result of the stimuli and experiences. The last criterion is the multi-sensory effect, which concerns the way in which stimuli are presented; the variety of stimuli and experiences proposed favors the learning experience. In a 2006 study, Feuerstein described the concept of cognitive modifiability as the individual’s ability to modify his or her responses through motor practice and experience. This capacity constitutes a condition of plasticity in an organism, which, as a result of motor and sporting activity, becomes capable of improving, changing and developing new learning strategies.

## 4. The Role of BDNF in Improving Learning and Memory

Underlying the relationship between motor activity and hippocampal neurogenesis is a molecule recognized as a key element in mediating this process, BDNF or the Brain-Derived Neutrophic Factor. BDNF, a polypeptide belonging to the neurotrophin family, regulates many of the processes within neurogenesis in the adult brain and is implicated in the mechanisms of synaptic plasticity, which are fundamental for the learning, survival and differentiation of neurons. By promoting synaptogenesis, BDNF regulates the number of synapses and makes neurons capable of developing stable variations in response to the synaptic stimuli they are subjected to [44]. This phenomenon underlies the plastic capacity of the brain and is responsible for complex phenomena such as learning and memory. The fact that BDNF plays a major role in the functionality of neurons implies that an increase in its expression through activity-dependent stimulation is necessarily and significantly correlated with an increased functionality of cognitive processes, determined by a higher degree and speed of learning [45]. By increasing BDNF synthesis, motor activity would induce the creation of an ideal neurobiological environment such that it becomes a significantly predisposed terrain for the changes and adaptations induced by the learning processes [46]. Constantly practiced motor activity improves hippocampal function and, consequently, performance in tasks requiring the use of spatial memory [47]. In 1996, Neeper et al. [48] demonstrated how motor activity in rodents was able to produce the increased expression of a gene regulating the production of the neurotrophin BDNF, which is responsible for the growth of the nervous system, the proper functioning of neurons and their defense against free radical damage. It can, therefore, be deduced that the more a mouse had run during its life, the greater its production of neurotrophin [49]. The cognitive aspects that would most seem to benefit from this increase in BDNF protein are memory and executive functions [50], as well as an improved performance related to attention, inhibitory control, work speed and visual learning [51,52,53]. A large area of study devoted to research on the possible links between motor activity and increased BDNF expression shows how both are associated with increased neurogenesis, especially at the level of the dentate gyrus of the hippocampus [54,55]. In laboratory tests on test subjects, it has been shown that increased BDNF can be observed after only a few days of exercise, and that such levels of increased Neurotrophic Factor can be maintained for several weeks [56,57,58,59]. The generation of new neurons and the increased plastic capacity of the hippocampal region make it possible to explain the improvement in cognitive abilities, as this area of the brain is responsible for memory consolidation and learning [54]. Consistent with these findings, higher levels of BDNF stimulated through motor activity, and consequently associated with increased synaptic plasticity and long-term neuronal potentiation, result in better memory and spatial recognition abilities that can also counteract the effects of senile cognitive decline [60,61,62,63,64,65,66]. However, in order to be able to achieve a significant improvement in learning abilities, a decisive factor would appear to be the administration of motor practice in terms of duration, frequency and intensity [67,68]. Nevertheless, it has been possible to observe that the best results can be achieved through moderate motor activity, especially when conducted over a prolonged period of time [69].

## 5. Aerobic Motor Activity

Research conducted over the past decade in both animal models and humans suggests that aerobic motor activity is the ideal delivery mode, as it is capable of elevating basal BDNF levels in the hippocampus above a statistically significant threshold [70,71,72,73,74,75] through the positive regulation of learning-related brain processes and the induction of functional and structural changes in the CNS. Following aerobic motor activity, the brain becomes more efficient and adaptable, and is able to positively modify its plasticity through the mechanisms of neurogenesis, neuroadaptation and neuroprotective processes. All this inevitably translates into improved memory and executive function through mechanisms that promote synaptic plasticity, neurogenesis and the generation of new brain cells capable of integrating into pre-existing neuronal networks [76,77].

Recent studies conducted on elderly populations have shown that aerobic motor activity is not only instrumental in improving various cognitive areas, but that it is also protective against cognitive decline, favoring the maintenance of memory, processing speed and executive function, traits that are relatively widespread in the ageing process [78,79,80,81,82]. This effect is due to the positive influence that motor activity exerts on various brain regions capable of generating increased serum levels of neurotrophins. Aerobic motor activity would create the most suitable conditions for making the CNS easily predisposed to the changes and adaptations necessary for learning new functions [83,84,85]. In this sense, it can be used both as enhancement training and as maintenance training for brain functions [86]. Aerobic physical activity would appear to induce improvements in the frontal brain areas especially [87]. The greatest positive effects have been observed on improvements in capacities related to executive functions, memory, attention, working memory, concentration, planning and processing speed; functions that are managed precisely by the prefrontal cortex. This selectivity of action of the training is confirmed by magnetic resonance images that allow the presence of increased grey matter in the frontal regions to be observed. These results can be interpreted as evidence in favor of increased neuronal efficiency during executive and memory tasks [88]. There are, therefore, some experimental data supporting the role of BDNF as a mediating factor for cognitive improvements; as the hippocampus is crucial for memory consolidation and learning, so is the generation of new neurons and the increased plasticity in this region [89,90]. The most current studies in the field of neuroscience applied to motor activity suggest that BDNF has numerous beneficial effects on the mind, as it is capable of inducing significant performance improvements on aspects such as attention and cognitive control, the emotional and social sphere, and short- and long-term memory [91,92]. In light of the numerous scientific evidence provided by the most current neuroscientific research, it is possible to state that BDNF could therefore be a potential modulator of the effects of aerobic motor activity on the cognitive performance of young adults.

## 6. Relationship between Anaerobic Exercise and Cognitive Function

At present, the greater part of the studies exploring the impact of physical exercise on cognitive function has focused on aerobic exercise. A significant number of meta-analyses that involve both aerobic and anaerobic exercise have found mild to moderate training-induced enhancements in different domains including processing speed, attention, executive function and memory [93,94,95,96,97,98,99,100,101,102].

Earlier in the development of the field, a number of investigations undertook a comparison of the impact of aerobic and resistance exercise on cognitive function, thereby revealing a potential dissimilarity in the effects yielded by these two exercise modalities [103]. Pennix et al. [104] aimed to conduct a more comprehensive investigation into the impact of physical activity on emotional states and overall physical health, with a specific focus on distinguishing potential disparities between aerobic and resistance exercises, if discernible. Participants in the aerobic group exhibited a pronounced decrease in depression symptom scores over time, in contrast to those in the control group. On the other hand, individuals in the resistance exercise group experienced a shift in symptoms. However, this alteration did not demonstrate any significant divergence from the shift observed in the control group. The presence of evidence indicating that aerobic exercise had a pronounced impact on working memory, in contrast to the absence of any discernible outcome in the resistance exercise group, signifies that aerobic and resistance exercise may diverge in their influence on cognitive function.

Recent studies [105,106,107] have also focused on investigating the potential influence of resistance exercise due to the observed favorable outcomes. Compared to the practice of endurance training, commonly characterized by the repetitive performance of a singular movement pattern such as running or cycling, resistance training frequently encompasses a sequence of diverse exercises targeting both the upper and lower extremities. Therefore, it is possible to hypothesize that strength training, due to its greater variability, could potentially elicit a comparable level of brain stimulation as aerobic exercise. The systematic reviews that are currently available have reported a range of results, with predominantly modest benefits, in relation to resistance exercise and its impact on various cognitive measures [108]. The results of this study align with the data presented in two meta-analyses that examined trials involving the recruitment of healthy older individuals for long-term training interventions. Kelly et al. [109] were unable to identify any impact of resistance exercise on working memory or attention, although they did observe a substantial enhancement in reasoning. Nevertheless, the size of their sample was relatively limited, as they only identified a maximum of three studies for each cognitive dimension. In 2018, Northey et al. [98] successfully aggregated the findings from thirteen trials. The researchers documented notable impacts of resistance exercise on cognitive processes such as executive function, memory and working memory. In comparison to the data accessible for long-term regimens, there is a paucity of evidence pertaining to the immediate consequences of a single training session.

Recently, Wilke et al. [110] provided a comprehensive overview of the existing body of evidence pertaining to the immediate impacts of resistance exercise on cognitive capabilities among the adult population. The findings of their study illustrate that a single session of training has the capability to elicit modest enhancements in performance. The postulated mechanisms through which physical activity might impact cognitive functioning could account for this discrepancy. It has been demonstrated that acute aerobic exercise has the potential to augment cerebral blood flow [111]. A comparable phenomenon might arise following resistance exercise. Nevertheless, while the primary factors influencing cerebral perfusion subsequent to aerobic exercise are neuronal exigency, cardiac output and the partial pressure of arterial carbon dioxide, it is plausible to surmise that resistance exercise could instead instigate fluctuations and/or surges in blood pressure, leading to alterations in blood flow [112]. An alternative aspect pertains to changes in the concentration of cortisol in the bloodstream.

To the best of our knowledge, the study by Sibley and Etnier [113] remains the only review that aims to evaluate the effects of anaerobic exercise on children. Nonetheless, it is considerably antiquated and has yet to present a comprehensive statistical summation of the gathered information.

## 7. Motor Activity and Cognitive Involvement

Without motor activity conducted with a positive relationship with the surrounding environment, an individual’s cognitive and social development would be severely compromised. If the experiences of the individual during movement are rich and different, more neuronal connections can be created and, consequently, more new skills can be learned by the individual [114]. Numerous studies suggest that cognitively engaging motor activity, i.e., those activities that adhere to a qualitative approach, can improve cognitive function. According to Tomporowsky and Pesce [115], this happens, plausibly, because qualitative physical activities that intentionally include problem-solving demands and lead to the acquisition of declarative and procedural knowledge, as well as immediate response strategies, could lead to the long-term development of abilities to exercise control over thinking and actions.

According to Vazou et al. [116], motor activity is able to have a positive influence on intellectual performance because many motor tasks are cognitively engaging. In particular, activities performed in a group encompass a great variety of cognitive demands that the individual must put in place in order to perform the required motor task. Specifically, open-skills sports, characterized by specific situations of contextual interference, allow the learning of new skills to occur more quickly and in a more lasting and reinforced manner [117]. Presumably, this occurs because the subject’s processing of information becomes more complex and demanding, leading to a higher degree of cognitive learning [118,119,120]. In high-performance motor and sporting activities, cognitive qualities are required that include speed of response to stimuli, high memorization and a rapid change of action in relation to situational changes, as well as temporal and motor anticipation skills. This complex of cognitive activities required by motor and sporting activity allows for a restructuring of brain maps and an improvement in mental functionality such that the learning experience is strengthened and enriched [121,122]. Motor and sports activity is, therefore, capable of stimulating the creation of new neuronal connections and reorganizing cortical maps to such an extent that learning processes become an effective and rewarding experience. In this sense, motor activity, with cognitively engaging tasks, is capable of inducing and emphasizing cognitive processes and is an effective tool for achieving a person’s full cognitive potential [123,124].

## 8. Discussion

Motor and sports activity can make brain functions more efficient, plastic and adaptable, which translates into a significant improvement in memory and executive function due to positive modifications that trigger changes in neurogenesis, synaptic plasticity and neuronal proliferation. The data discussed in this review show that motor activity can be used to improve cognitive abilities both in healthy individuals and in those with cognitive decline, as it would be a predisposing terrain on which to accommodate the changes and adaptations induced by learning new and more evolved functions and skills. Research in the field of neuroscience points to a profound interdependence between movement and cognitive processes. According to a conceptualization model proposed by many authors [125,126,127,128], this interdependence is mainly related to the changes that occur in brain function and structure as a consequence of motor activity. In recent years, research in this field has evolved considerably, through studies born out of the need to better understand the multifaceted relationship between motor activity and cognitive health. Advances in neuroscience and neuroimaging techniques have allowed researchers to better understand the effects of motor activity on cognitive function by investigating its physiological and molecular mechanisms [129,130,131]. Research conducted in the different areas of interest regarding motor activity has helped to define this line of investigation and to identify some preliminary underlying mechanisms. This has resulted in a deeper understanding of the components that are linked to cognitive function and that may be amenable to intervention [132,133]. Investigations into the influence of motor activity on cognitive functioning have proposed several mechanisms that could explain this relationship [134,135]. Numerous studies published in recent years on this topic have mainly concluded that motor activity is able to bring about a series of biological and structural modifications and adaptations that allow brain cells to create new connections in different cortical areas. In fact, motor activity is able to trigger a cascade of neurochemical growth factors capable of changing the entire brain’s structure. This reflects the brain’s ability to adapt to the various cognitive challenges it faces [136,137]. However, until recently, the relationship between intensive exercise and cognitive function was not entirely established, in light of the fact that the literature on the subject seemed to provide somewhat contradictory results. Indeed, while a number of studies indicated that short sessions of motor activity could induce benefits in terms of cognitive functioning, others found no such beneficial effect [138,139].

**Figure 1:**
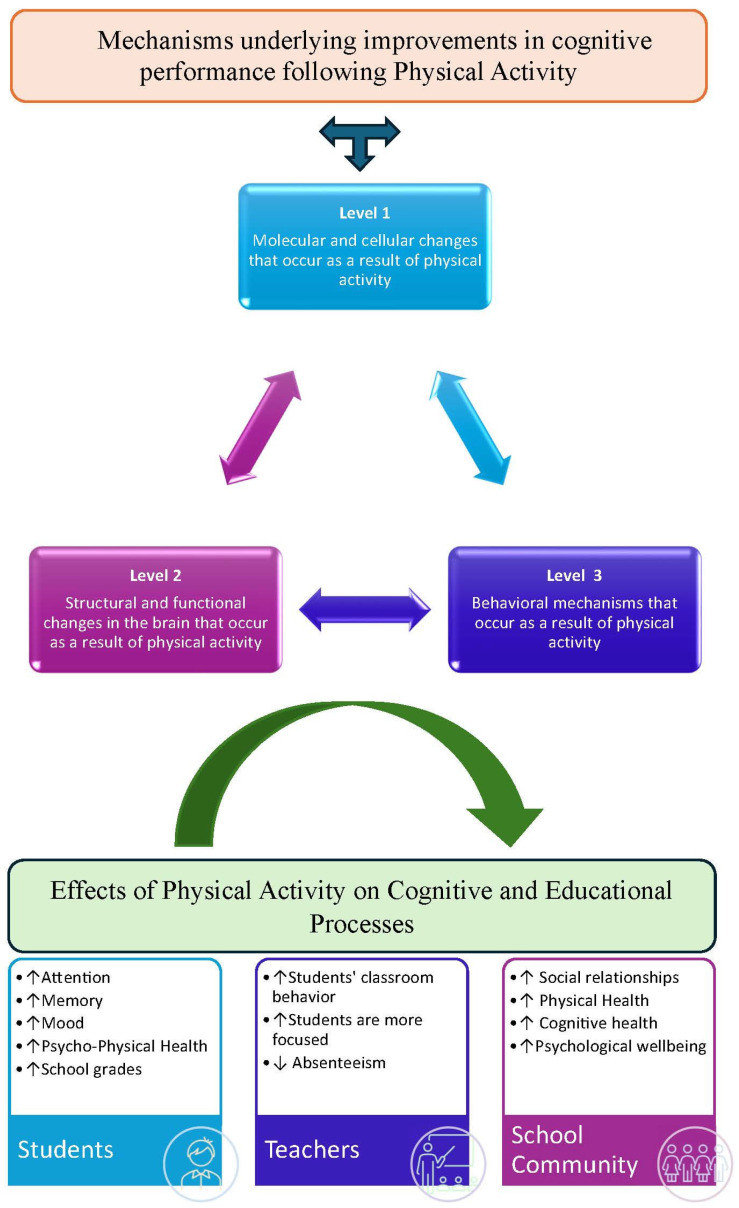
Effects of physical activity on cognitive function.

In more recent times, it has been shown that the effect of motor activity on cognitive performance depends on both the intensity and duration of the exercise, as well as the intrinsic aspects that characterize the proposed activity, i.e., the cognitive engagement required by the exercise [140]. Although it would appear that the most fruitful mode of motor activity delivery may be that of moderate-intensity aerobic exercise, and that the first hour after the end of exercise itself may be the most likely window of time within which motor learning processes are most facilitated, research in recent years has focused on understanding how motor activity with cognitive involvement is most likely to influence cognitive development and learning processes [141]. The most recent scientific evidence shows, therefore, how the effects of motor activity on cognitive abilities can lead to favorable results in terms of improving both low-level cognitive functions related to simple reactions and higher-level functions such as memory, processing speed, attention and executive function [90,117]. Overall, the results of these studies suggest that motor activity is positively correlated with children’s cognitive functioning and learning abilities, but these relationships are not universal and vary depending on the outcome studied and the different variables taken into account Figure 1.

## 9. Conclusions

The modern neuroscientific approach toward the relationships between motor activities and cognitive functions suggests that hypokinesia tends to inhibit learning. In this context, neuroscience applied to motor and sporting activity forces us to pay particular attention to the growing number of children and adolescents with learning disorders. Therefore, it now seems more topical than ever to draw attention to the need to introduce working proposals that integrate brain-based motor activity programs into the school curriculum. Future research collaborating with educational institutions should, therefore, allow us to work together in order to maximize the use of adapted physical activity within the regular curriculum.

## Data Availability

The data presented in this study was obtained from the included studies and was openly available.
